# 晚期肺神经内分泌肿瘤免疫检查点抑制剂治疗进展

**DOI:** 10.3779/j.issn.1009-3419.2021.102.42

**Published:** 2021-11-20

**Authors:** 浩庆 陈, 庆威 孟

**Affiliations:** 150000 哈尔滨，哈尔滨医科大学附属肿瘤医院肿瘤内科 Department of Medical Oncology, Harbin Medical University Cancer Hospital, Harbin 150000, China

**Keywords:** 肺神经内分泌肿瘤, 免疫检查点抑制剂, 生物学标志物, 免疫治疗, Pulmonary neuroendocrine tumors, Immune checkpoint inhibitors, Biomarkers, Immnotherapy

## Abstract

肺神经内分泌肿瘤（pulmonary neuroendocrine tumors, PNETs）是一种源于肺神经内分泌细胞的上皮性肿瘤，约占原发性肺肿瘤的20%，包括典型类癌、非典型类癌、小细胞癌和大细胞神经内分泌癌。这四类PNETs之间的形态学和临床特征具有较大的异质性。免疫检查点抑制剂（immune checkpoint inhibitors, ICIs）已在多种实体瘤中被证实具有很强的抗肿瘤活性。晚期PNETs治疗方案在过去十年内已有了很大的发展，但ICIs在PNETs领域的应用才刚刚起步。本文主要阐述目前PNETs中ICIs临床试验和研究的格局以及免疫检查点抑制剂治疗相关标志物的现状。

## Abstract (en)

Pulmonary neuroendocrine tumors (PNETs) are a kind of epithelial tumors originating from pulmonary neuroendocrine cells, accounting for about 20% of primary lung tumors, including typical carcinoid, atypical carcinoid, small cell carcinoma, and large cell neuroendocrine carcinoma. The morphologic and clinical characteristics of these four types of PNETs are relatively highly heterogeneous. Immune checkpoint inhibitors (ICIs) have been shown robust antitumor activity in a variety of solid tumors. Treatment regimens of advanced PNETs have developed greatly in the past decade, but ICIs are still in their infancy in the field of PNETs. This review focuses on the landscape of current clinical trials and research as well as the situation of ICIs-related biomarkers in PNETs.

肺神经内分泌肿瘤（pulmonary neuroendocrine tumors, PNETs）是一种源于肺神经内分泌细胞的上皮性肿瘤，具有独特的生物学和临床学特征，约占原发性肺肿瘤的20%，是神经内分泌肿瘤（neuroendocrine neoplasms, NENs）的常见类型^[1]^。美国国立癌症研究所数据库（Surveillance, Epidemiology, and End Results, SEER）显示，NENs年发病率逐年增加，由1973年的1.09/10万人升高到2012年的6.98/10万人。其中PNETs年发病率增加了4.36倍，自1973年的0.35/10万人升高到2012年的1.62/10万人^[2]^。

过去十年来已有许多治疗方式（如生长抑素类似物、肽受体放射性核素治疗、依维莫司、化疗以及抗血管生成药物）用于晚期PNETs的临床治疗中^[3]^。生长抑素类似物适用于PNETs合并类癌综合征，肽受体放射性核素治疗适用于生长抑素受体阳性的PNETs患者，依维莫司适用于非功能性PNETs，适用于PNETs的化疗药物包括链脲菌素、铂类、依托泊苷及替莫唑胺，抗血管生成药物在PNETs中也被认为有潜在疗效。

免疫检查点抑制剂（immune checkpoint inhibitors, ICIs）已经在多种实体瘤中获得了革命性进展，但ICIs在PNETs领域的作用仍有待探索。本文围绕近年来PNETs的ICIs治疗的现状和进展，作一综述。

## 1 PNETs分类

根据2015年世界卫生组织分类标准，PNETs分为典型类癌（typical carcinoid, TC）、非典型类癌（atypical carcinoid, AC）、小细胞癌（small cell lung carcinoma SCLC）和大细胞神经内分泌癌（large cell neuroendocrine carcinoma, LCNEC）^[4]^。

四类PNETs之间的生物学特征具有较大的异质性，可分为高级别PNETs和低中级别PNETs。TC和AC属于高分化的低中级别PNETs，患者年龄较小，预后更好，与吸烟史无密切联系，很少有基因突变^[4]^。TC的有丝分裂率小于2个/2 mm^2^且无坏死，AC有丝分裂率则为2个/2 mm^2^-10个/2 mm^2^^[3]^。SCLC和LCNEC属于低分化的高级别PNETs，通常表现为高增殖活性、高有丝分裂率（≥10个/2 mm^2^）、广泛坏死。SCLC和LCNEC与吸烟史有密切联系，两者具有相互重叠的基因突变，常见的基因突变包括*TP53*、*RB1*、*MYC*/*MYCL1*、*MLL2*、*LRP1B*和*PTEN*等^[1, 5, 6]^。目前SCLC的ICIs临床试验较多，IMpower-133^[7]^和CASPIAN^[8]^等临床试验证实了ICIs联合化疗一线治疗可有效改善患者总生存期，但LCNEC和类癌的ICIs临床试验目前研究较少，因此本文主要围绕LCNEC和类癌进行阐述。

## 2 免疫逃逸机制

肿瘤免疫逃逸的一个重要机制是通过免疫检查点以阻断特异性T细胞特异性免疫反应。正常细胞中，T细胞对抗原的识别及效应功能受到免疫检查点的严格调控，以防止外周组织过度免疫反应^[9]^。目前研究最为深入的免疫检查点有两个，其中一个免疫检查点由程序性死亡配体1（programmed death ligand 1, PD-L1）和程序性死亡蛋白1（programmed death protein 1, PD-1）组成，另一个免疫检查点由细胞毒性T淋巴细胞相关蛋白-4（cytotoxic T lymphocyte-associated protein-4, CTLA-4）和共刺激配体B7-1及B7-2组成。当肿瘤细胞表达的PD-L1和效应T细胞表面的PD-1结合时，T细胞增殖抑制和抑制性细胞因子分泌增多，从而逃避T细胞介导的杀伤作用。CTLA-4属于T细胞上的跨膜受体。肿瘤可以通过诱导微环境下CTLA-4表达上调，导致免疫检查点发挥作用并下调T细胞免疫反应。因此ICIs与相应的检查点分子结合后阻断免疫逃逸机制，逆转被抑制的免疫反应，恢复细胞毒性T细胞免疫活性^[10]^。目前常见的ICIs包括CTLA-4抑制剂如Ipilimumab和Tremelimumab，PD-L1抑制剂如Avelumab、Durvalumab和Atezolizumab和PD-1抑制剂如Nivolumab、Pembrolizumab和Spartalizumab。

## 3 ICIs相关生物学标志物

### 3.1 PD-L1表达

PD-L1为一种跨膜蛋白，通过与免疫细胞表面PD-1结合调节免疫细胞活性。通常情况下，肿瘤细胞利用PD-L1和PD-1的相互作用，下调T细胞增殖并促进抑制细胞因子的产生来抑制抗肿瘤免疫反应^[11]^。因此，阻断PD-L1/PD-1通路已成为目前各类恶性肿瘤热门的治疗方案之一。近年来，多项非小细胞肺癌（non-small cell lung cancer, NSCLC）临床试验已确认PD-L1表达阳性为筛选ICIs获益人群的有效生物学标志物。KEYNOTE-001研究^[12]^显示PD-L1≥50%与ICIs疗效改善有关，KEYNOTE-024临床研究^[13]^显示，与化疗相比，PD-L1≥50%的晚期NSCLC患者使用Pembrolizumab可显著延长无进展生存期（progression-free survival, PFS）和总生存期（overall survival, OS）。ATLANTIC和POPLAR研究也揭示了PD-L1表达阳性和ICIs预后改善的显著关系^[14, 15]^。因此探究PNETs的PD-L1表达对于临床运用ICIs具有一定的指导意义。

目前研究^[16, 17]^显示四种PNETs的PD-L1表达水平有很大的差异性，其阳性表达率在16%-85%之间。这种差异可能与不同研究使用的抗体和截断值不一样有关。AC的PD-L1表达情况极低，几乎全部为阴性表达，TC被报道有7%的PD-L1阳性率^[16, 18, 19]^，SCLC和LCNEC的PD-L1阳性率比类癌高，分别为39%和75%^[20, 21]^。

### 3.2 肿瘤突变负荷（tumor mutational burden, TMB）

TMB是每百万碱基中被检测出的细胞基因突变总数，是反映肿瘤细胞携带基因突变总数的一种定量生物学标志物。TMB越高预示肿瘤表面能够刺激免疫反应的抗原越多，肿瘤细胞被毒性T细胞识别并杀伤的概率增加。多项NSCLC临床研究提示高TMB与ICIs预后和疗效改善有关。CheckMate 026研究^[22]^中Nivolumab一线治疗晚期NSCLC的结果显示，高TMB患者的免疫治疗应答率高于化疗组（47% *vs* 28%），无进展生存期更长（中位生存期9.7个月*vs* 5.8个月）。CheckMate 227^[23]^和CheckMate 568^[24]^研究也表明高TMB（≥10 mut/Mb）是ICIs临床预后获益的独立预测因素。TMB对PNETs患者ICIs治疗是否具有类似的预测能力，仍有待于研究。

目前少数研究表明PNETs的总体TMB表达水平较高。Chae等^[6]^的回顾性研究显示SCLC和LCNEC患者的中位TMB均为9.9 mut/Mb，LCNEC的TMB上下四分位数分别为4.5 mut/Mb和17.1 mut/Mb，SCLC的TMB上下四分位数分别为6.3 mut/Mb和14.4 mut/Mb。Chi等^[25]^的回顾性研究认为PNETs的中位TMB为11.0 mut/Mb，Sabari等^[26]^回顾性研究显示LCNEC的中位TMB明显高于SCLC（15.3 mut/Mb *vs* 8.2 mut/Mb）和NSCLC（15.3 mut/Mb *vs* 5.7 mut/Mb）。

## 4 ICIs治疗现状和进展

### 4.1 LCNEC ICIs治疗（表 1）

**表 1:** LCNEC相关免疫治疗回顾性研究及临床研究 ICIs related retrospective study and clinical trial for LCNEC

Research	Phase	*n*	Drugs	ORR	mPFS (mon)	mOS (mon)	Identifier
Matteo *et al*^[27]^	Retrospective	10	pem or nivo	60%	13.3		
Shira *et al*^[28]^	Retrospective	21 *vs* 270*	ICIs	33%	4.2	11.8 *vs* 6.9*	
DART SWOG 1609^[33]^	II	32	ipi+nivo	25%	4.0	11.0	NCT02834013
LCNEC: large cell neuroendocrine carcinoma; ICIs: immune checkpoint inhibitors; ORR: objective response rate; mPFS: median progression-free survival; mOS: median overall survival; nivo: Nivolumab; pem: Pembrolizumab; ipi: Ipilimumab; *: non-LCNEC group; mon: month.							

LCNEC的ICIs治疗证据主要来源于回顾性研究和病案报告。法国一项多中心回顾性研究^[27]^评估了Nivolumab或Pembrolizumab用于化疗后进展晚期LCNEC的ICIs疗效。纳入的10例患者中，有8例患者为IV期疾病，中位年龄为59岁。使用ICIs后60%的患者部分缓解，10%的患者疾病稳定，中位无进展生存期（median PFS, mPFS）为13.3个月。10例患者中仅有1例在检测PD-L1阳性后使用Pembrolizumab，其余9例未经PD-L1检测即接受了Nivolumab治疗，因此无法进一步验证PD-L1能否筛选潜在获益人群。

另一项回顾性研究^[28]^进行了晚期LCNEC患者和非LCNEC晚期肺癌患者单药ICIs疗效评估。研究结果显示，LCNEC组mPFS为4.2个月，客观缓解率（objective response rate, ORR）为33%，11%的患者出现完全缓解，22%的患者出现部分缓解；LCNEC组患者较非LCNEC组患者中位总生存期（median OS, mOS）占优（11.8个月*vs* 6.9个月）。该研究未观察到OS和肿瘤PD-L1表达的显著相关性，这可能和LCNEC的罕见性和临床样本量少有关。

除外回顾性研究，诸多病例报道显示出ICIs在LCNEC上具有显著临床活性。1例不可切除的局部晚期LCNEC的41岁女性患者在含铂化疗3个周期后出现肿瘤进展，行姑息性胸部放疗后，使用Nivolumab单药治疗获得显著疗效（PD-L1表达 > 1%），用药8个周期和14个周期观察到了肿瘤持续消退。最后，该例患者进行了挽救性手术，术后8个月内一直处于肿瘤完全缓解状态^[29]^。该例患者ICIs疗效优异的潜在机制可能是肿瘤细胞放疗后释放大量肿瘤抗原，联合ICIs后触发了更持久的免疫反应。提示ICIs联合放疗很可能具有协同作用，是很有希望的一种LCNEC治疗模式。Chauhan等^[30]^报道了3例LCNEC患者ICIs治疗情况，1例合并多发转移患者6个周期化疗后使用Nivolumab，出现原发灶缩小且肝脏转移疾病稳定；另外1例合并脑转移患者二线Nivolumab治疗后获得完全缓解；第3例患者伴有高TMB和PD-L1阳性，ICIs治疗后呈现疾病稳定。Takimoto等^[31]^报道了1例IVb期LCNEC老年男性患者Nivolumab三线治疗情况，尽管在2个周期后由于间质性肺炎而停止治疗，患者疾病稳定约6个月且期间无新增免疫相关不良反应。另外有病例报道称1例早期LCNEC患者免疫组化显示PD-L1阴性，基因检测显示*PD*-*L1*扩增、*MYC*扩增、*STK11*（*LKB*）、*APC*、*RB1*和*TP53*等基因突变以及高TMB（24.76 mut/Mb），该患者在Pembrolizumab治疗1个周期后肿瘤病灶全部缩小，并获得6个月疾病持续稳定^[32]^。

除外免疫单药治疗，不同机制的ICIs联合治疗可能是一种潜在的选择。II期临床试验DART SWOG 1609研究纳入了32例NENs患者，其中6例患者为PNETs，入组患者接受Nivolumab和Ipilimumab双药治疗。总体ORR为25%，高级别NENs的ORR为44%，低中级别NENs的ORR为0%。总体6个月PFS率为31%，高级别NENs组6个月PFS率为44%，低中级别NENs则为14%^[33]^。该研究结果提示在高级别LCNEC中使用免疫双药可能具有更好的ORR。然而，38%的患者发生了3级-4级免疫治疗相关不良事件，31.5%的患者因不良事件停止了ICIs治疗，因此使用免疫双药时应注意对免疫治疗相关不良事件的管理。

与SCLC相比，LCNEC免疫用药仍缺乏前瞻性随机对照试验的证据支持。目前有少量LCNEC前瞻性临床试验正在开展，如Pembrolizumab联合铂类和依托泊苷化疗一线治疗晚期LCNEC的II期临床试验（NCT03901378），以及Nivolumab联合Ipilimumab二线/三线以上治疗用于转移性或不可切除的复发性LCNEC的II期临床试验（NCT03591731）。

### 4.2 类癌ICIs治疗（表 2）

**表 2:** 类癌相关免疫治疗临床试验及回顾性研究 ICIs related clinical trials and retrospective study of immunotherapy for carcinoid

Research	Phase	*n*	Drugs	ORR	mPFS (mon)	mOS (mon)	Identifier
KEYNOTE-028^[35]^	Ib	107	pem	12.0%	5.6	21.1	NCT02054806
Janice *et al*^[36]^	Retrospective	25	pem	12.0%	5.6		
Yao *et al*^[38]^	II	30	spa	16.7%			NCT02955069
CA209-538^[39]^	II	29	ipi+nivo	24.0% (AC: 33.0%)	4.8	14.8	NCT02923934
spa: Spartalizumab; AC: atypical carcinoid.							

早期类癌首选手术，晚期类癌治疗目前仍缺乏国际公认的标准方案^[34]^。KEYNOTE-028研究^[35]^是一项非随机Ib期临床试验，纳入了475例PD-L1阳性且接受Pembrolizumab治疗的晚期实体瘤患者，其中25例为PD-L1阳性晚期类癌患者。类癌亚组试验结果显示，ORR为12%，15例患者疾病稳定，mPFS及mOS分别为5.6个月和21.1个月。该研究还显示PD-L1与ORR（*P*=0.018）及PFS（*P*=0.005）之间存在显著相关性，高TMB也与ORR（*P*=0.018）以及PFS（*P*=0.051）改善有关。基于KEYNOTE-028结果，Mehnert等^[36]^评估了Pembrolizumab对PD-L1阳性晚期类癌或胰腺NENs的疗效，其中类癌队列有25例患者接受Pembrolizumab单药治疗。该试验纳入患者均为PD-L1表达阳性，类癌队列ORR为12.0%（95%CI: 2.5%-31.2%），有3例患者获得部分缓解，32%患者疾病稳定超过6个月，mPFS为5.6个月，中位缓解持续时间为9.2个月。

Spartalizumab是一种高亲和力人源化的新型抗PD-1抗体，可同时阻断PD-L1和PD-L2与PD-1的结合。Yao等^[37]^开展的II期临床试验评估了Spartalizumab二线治疗对于晚期NENs的作用。纳入的116例患者中有30例为类癌患者。初步结果显示，分化良好NENs总ORR为7.4%，类癌ORR达到20%，初步结果显示了Spartalizumab在类癌中的临床活性。近来Yao等^[38]^对Spartalizumab的进一步研究显示，类癌队列ORR为16.7%，且PD-L1表达阳性患者和较高ORR有关，这预示着PD-L1在类癌ICIs治疗中可能具有预测意义。

II期临床研究CA209-538探索了Nivolumab和Ipilimumab双药治疗NENs的临床疗效。纳入的29例晚期NENs患者中，16例为中低级别NENs，13例为高级别NENs，其中PNETs占比最高，达38%。研究结果显示总体ORR为24%，临床获益率为72%，AC组ORR为33%，其中有2例患者获得完全缓解；总体mPFS和mOS分别为4.8个月和14.8个月。66%的患者出现免疫相关不良事件，34%的患者出现3级-4级不良事件，17%的患者由于3级-4级不良事件停止ICIs治疗^[39]^。该研究中AC使用免疫双药获得了较好的缓解率，提示免疫双药是AC治疗的潜在选择。

## 5 展望

ICIs治疗在许多癌症类型中都显示了巨大进展，目前被探索用于PNETs中。PNETs前瞻性临床试验极少，这和PNETs发病率较低、具有较大异质性有关。现有的研究主要涉及ICIs单药治疗，仅有少数患者能从中获益。未来相关ICIs临床试验应探索更多免疫联合治疗方式，如免疫双药、免疫联合化疗、免疫联合放疗以及免疫联合抗血管生成药物等。ICIs相关生物学标志物有待深入探索，以筛选出合适的患者。PD-L1和TMB是主要的预测性标志物，但对ICIs疗效的预测能力并不明确，联合PD-L1、TMB以及其他潜在标志物进行联合预测是未来ICIs精准治疗的方向。
